# Presence and severity of migraine is associated with development of primary open angle glaucoma: A population-based longitudinal cohort study

**DOI:** 10.1371/journal.pone.0283495

**Published:** 2023-03-24

**Authors:** Kyoung Ohn, Kyungdo Han, Jung Il Moon, Younhea Jung

**Affiliations:** 1 Department of Ophthalmology, Yeouido St. Mary’s Hospital, College of Medicine, The Catholic University of Korea, Seoul, Republic of Korea; 2 Department of Statistics and Actuarial Science, Soongsil University, Seoul, Republic of Korea; BaekSeok University, REPUBLIC OF KOREA

## Abstract

**Purpose:**

To examine the association between the presence and severity of migraine and development of primary open-angle glaucoma (POAG) using a nationwide population-based longitudinal cohort data.

**Methods:**

Data were retrieved from the Korean National Health Insurance Service for 2,716,562 individuals aged ≥ 40 years and assessed for the development of POAG from 2009 through 2018. Subjects were classified into the following 3 groups: healthy control subjects, subjects with mild migraine, and those with severe migraine. Hazard ratios (HR) of glaucoma development were calculated for each group. Subgroup analyses of subjects stratified by age, sex, lifestyle factors (smoking, drinking, and body mass index (BMI)), and comorbidities (diabetes, hypertension, and dyslipidemia).

**Results:**

During the 9-year follow-up period, the incidence rate of POAG per 1000 person-years was 2.41 and 3.25 in subjects without and with migraine, respectively. Among the migraine group, the incidence rate was 3.14 and 3.89 in mild and severe subgroups, respectively. The HR was 1.355 (95% CI, 1.300–1.412) and 1.188 (95% CI, 1.140–1.239) before and after adjusting for potential confounding factors in the migraine group per se. Regarding the severity of migraine, the adjusted HRs were 1.169 (95% CI, 1.117–1.224) in the mild migraine group, and 1.285 (95% CI, 1.166–1.415) in the severe migraine group compared to the control group. The results were consistent in subgroup analyses after stratifying by age, sex, lifestyle factors, and comorbidities.

**Conclusions:**

Migraine is associated with increased risk of POAG development. Furthermore, chronic and severe migraine is associated with greater risk of POAG development.

## Introduction

Migraine affects more than 10% of the adult population worldwide and it ranks among the world’s most disabling medical illnesses [1]. In addition, the economic and societal effect of migraine is substantial: it affects patients’ quality of life and impairs work, social activities, and family life [2–4]. Although the nature and mechanism of migraine are complex and remain incompletely understood, potential mechanisms include vasospasm, vascular endothelium-related hypercoagulability, and vascular changes related with cortical spreading depression [5, 6]. Migraine is considered a systemic vasculopathy and is associated with ischemic heart disease, stroke, and other cardiovascular diseases [6].

Glaucoma is also a multifactorial disease characterized by progressive optic neuropathy and distinctive visual field loss [7, 8]. While intraocular pressure has been identified as the most important risk factor for its development, other risk factors have been identified [9, 10]. It has been known that female [11], older age [8, 12, 13], smoking [14, 15], drinking [14, 16], underexercising [14, 17], lower BMI [14, 18–20], and CKD [21] increase the risk for glaucoma. Both systemic vascular factors, such as hypertension and diabetes, and ocular vascular factors, such as ocular blood flow and ocular perfusion pressure, have been identified as risk factors, emphasizing the role of vascular mechanisms in its pathophysiology [9, 10, 22].

This association seems even stronger in those with normal tension glaucoma (NTG) [23], which is the most prevalent type of glaucoma in Korea. The proportion of NTG among POAG patients in Korea was 77% in the Namil epidemiologic study [24]. Therefore, we included the patients with NTG into the subject population of the present study to better represent the epidemiologic situation in Korea.

Considering this common etiology, a potential association between migraine and primary open angle glaucoma (POAG) has been previously studied, but the results are inconclusive. The Blue Mountains Eye Study demonstrated a positive association between migraine and POAG among those aged 70–79 [25]. In contrast, the Beaver Dam Eye Study found that there was no evidence of a relationship between open-angle glaucoma and migraine [26]. In a meta-analysis investigating the association between migraine and glaucoma, a significant association was found in case-control design studies, but not in cohort design studies, and the authors concluded the association is still controversial [27]. In addition, no study has explored the risk of glaucoma according to the severity of migraine.

Therefore, this study investigated the association between migraine and increased risk of developing POAG over a 9-year follow-up period using a nationwide longitudinal cohort data. We also examined whether this potential association was proportional with the chronicity and the severity of migraine.

## Methods

### Database

Data were retrieved from the Korean National Health Insurance Service (KNHIS) database, namely, the National Health Information Database (NHID) and the Korean Health Examination Database (KHED). All Koreans residing in the Republic of Korea are obliged to join the KNHIS since 1989. Collectively, the NHID contains medical data (e.g., personal information, diagnosis, medical treatment), history of comorbid diseases (e.g., hypertension, dyslipidemia, stroke, diabetes mellitus), and demographics (e.g., age, sex, household income) of patients. In addition, KNHIS also provides a standardized health screening program to all Koreans aged 40 or more. The KHED includes anthropometric data and laboratory tests. The KNHIS provides de-identified data to medical researchers who meet the access criteria.

This study was approved by the Institutional Review Board of Yeouido St. Mary’s Hospital, Seoul, Korea (SC21ZISE0175). Informed consent was waived by the IRB. This study was a retrospective cohort study, and all data were fully anonymized by the KNHIS before assessed by researchers. This study adhered to the Declaration of Helsinki.

### Exposure determination and outcome measurement

The exposure of interest was migraine diagnosis. Diagnosis for migraine was defined as at least one KNHIS claim under International Classification of Diseases (ICD)-10 code for migraine (G43) in 2009 (index year). Regarding the severity of migraine, subjects without chronic and severe migraine were further stratified into the “mild” group and those with chronic and severe migraine were categorized into the “severe” group. Chronic migraine was defined as diagnosis of migraine more than twice at least 3 months apart. Severe migraine was defined as follows: i) at least one hospitalization for migraine as the main diagnosis, ii) at least one visit to the emergency room for migraine as the main diagnosis, iii) Triptans (N02CC) and ergots (N02CA) are administered at least once within 1 year from the time of diagnosis, iv) perform the following procedures at least once within 1 year from the time of diagnosis: MM070, LA210, LA222, LA223, LA224, LA225, LA226, LA227, LA228, S0471, S0472, S0474, S0475, S0476, S0477, S0478, S4730, SZ636, SZ637, SZ638, or SZ639.

The outcome of interest was POAG. Development of POAG was defined as KNHIS claims with ICD-10 code for POAG (H401). The ICD-10 code H401 includes POAG and NTG. To strengthen the diagnostic validity, only those with at least three outpatient visits for glaucoma a year were included [28]. To establish a temporal and causal relationship between migraine and POAG, those with prior diagnosis of POAG before the index year and those who were diagnosed within one year after migraine diagnosis were excluded.

### Statistical analysis

The Student’s T-test was used to compare continuous variables between groups, and the Chi-square test was used to compare categorical variables. Cox’s proportional hazard regression was performed to estimate the hazard ratios for various risk factors. Hazard ratio for exposure of interest, history of migraine, was calculated using three models: model-1, un-adjusted; model-2, adjusted for age, sex, smoking habits, drinking habits, frequency of exercise, household income; model-3, adjusted for comorbid disease status (diabetes, hypertension, dyslipidemia, body mass index, and glomerular filtration rate), along with all risk factors addressed in model-2. Incidence probabilities of POAG to migraine was calculated using Kaplan-Meier survival analysis. All statistical analyses were performed using SAS (Version 9.4; SAS Institute, Cary, NC, USA). *p*-values ≤ 0.05 were considered statistically significant.

## Results

### Study population

**Fig 1:**
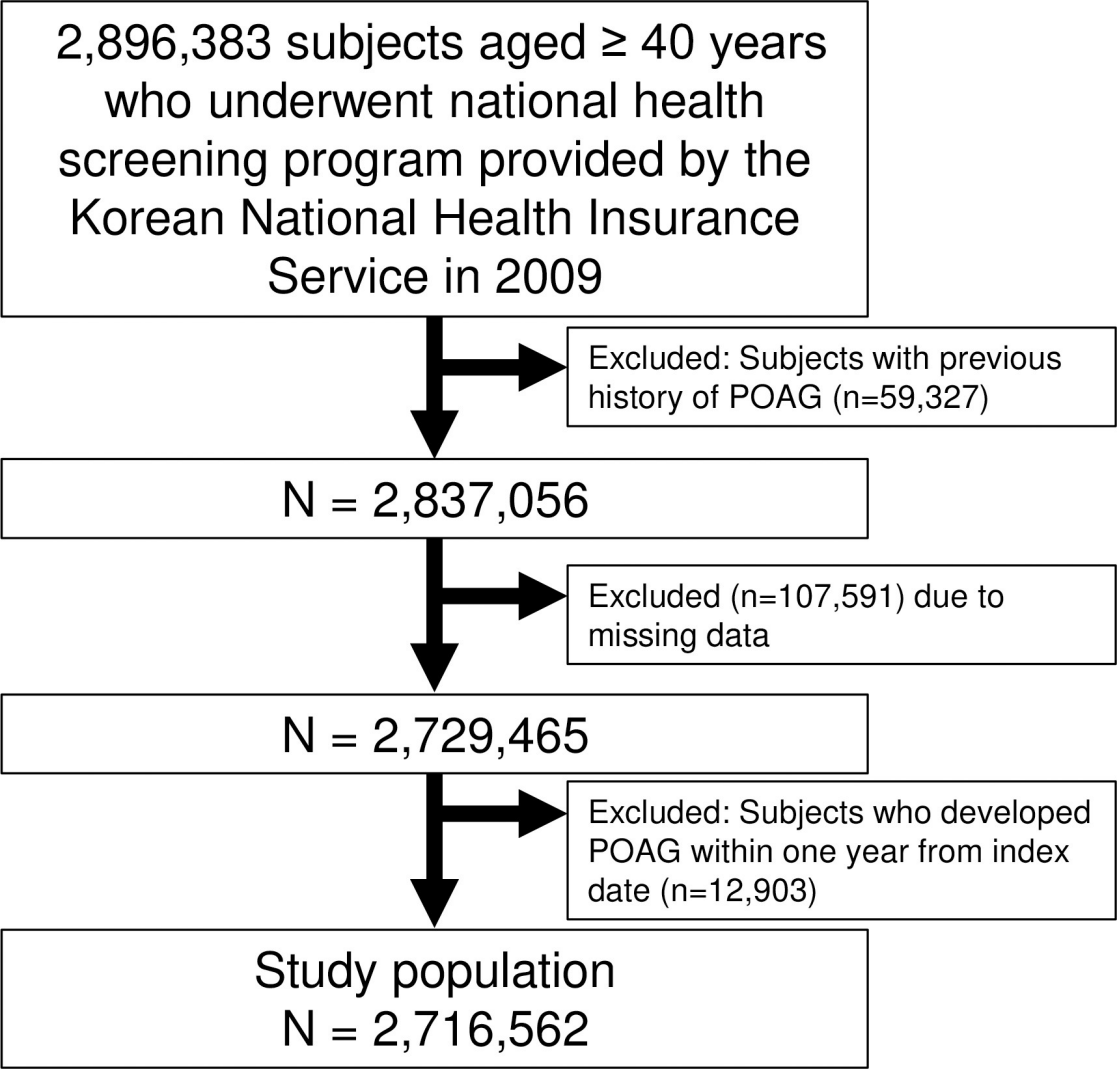
Selection of study population.

Fig 1 shows our study population. We first identified 2,896,383 individuals aged 40 years or more who had undergone national health screening program in 2009. After exclusion of 59,327 individuals who had a previous history of POAG, additional 107,591 individuals were excluded due to missing data. In addition, we excluded patients who developed POAG within one year from the index date. Finally, 2,716,562 individuals were enrolled and assessed for the development of POAG until 2018.

Table 1 presents the demographic characteristics, medical conditions, and laboratory test results of subjects in study population. Compared to subjects without history of migraine, subjects diagnosed with migraine were more likely to be female, non-smoker, non-drinker, had higher prevalence of hypertension, dyslipidemia, myocardial infarction, chronic heart failure, and stroke. Apart from that, we found small but statistically significant differences in age, sex, exercise, income, glomerular filtration rate (GFR), body mass index (BMI), waist circumference, systolic/diastolic blood pressure, and blood level of glucose, total cholesterol, high/low density lipoprotein, triglyceride, γ-glutamyl transpeptidase, aspartate transaminase, and alanine transferase between those with and without migraine.

**Table 1:** Demographic characteristics of study population.

		Migraine, n(%)		p-value
		No	Yes	
Parameters		2,628,753	87,809	
Sex, male		1,343,046 (51.09)	24,289 (27.66)	< .0001
Smoking status				< .0001
	Non	1,651,662 (62.83)	69,972 (79.69)	
	Ex-Smoker	414,682 (15.77)	8,376 (9.54)	
	Current	562,409 (21.39)	9,461 (10.77)	
Drinking status				< .0001
	Non	1,521,777 (57.89)	64,916 (73.93)	
	Mild	910,785 (34.65)	19,682 (22.41)	
	Heavy	196,191 (7.46)	3,211 (3.66)	
Regular exercise		529,358 (20.14)	15,980 (18.2)	< .0001
Monthly income, lower 25%		469,348 (17.85)	16,572 (18.87)	< .0001
Diabetes		303,486 (11.54)	9,836 (11.2)	0.0017
Hypertension		919,150 (34.97)	37,515 (42.72)	< .0001
Dyslipidemia		595,416 (22.65)	25,500 (29.04)	< .0001
Myocardial infarction		10,901 (0.41)	506 (0.58)	< .0001
Chronic heart failure		21,294 (0.81)	1,410 (1.61)	< .0001
Stroke		54,308 (2.07)	4,978 (5.67)	< .0001
Migraine				
	Chronic	0 (0)	19,987 (22.76)	< .0001
	Severe	0 (0)	49,387 (56.24)	< .0001
Migraine Group				< .0001
	Non	2,628,753 (100)	0 (0)	
	Mild	0 (0)	74,795 (85.18)	
	Severe	0 (0)	13,014 (14.82)	
Age, year		54.14±10.4	56.46±10.91	< .0001
GFR, mL/min/1.73m^2^		84.91±37.51	83.77±30.84	< .0001
Body mass index, Kg/m^2^		23.97±3.03	23.99±3.1	0.0769
Waist Circumference ratio, %		81.24±8.89	80.49±9.4	< .0001
Total cholesterol, mg/dl		199.34±42.04	201.56±45.76	< .0001
Blood glucose		100.01±25.91	98.58±23.39	< .0001
Systolic BP		124.22±15.52	123.97±15.6	< .0001
Diastolic BP		77.21±10.23	76.89±10.08	< .0001
HDL, mg/dl		56.16±34.12	56.82±35.21	< .0001
LDL, mg/dl		119.11±83.6	121.8±82.94	< .0001

### The risk of POAG among the study subjects during the 9-year follow up

**Fig 2:**
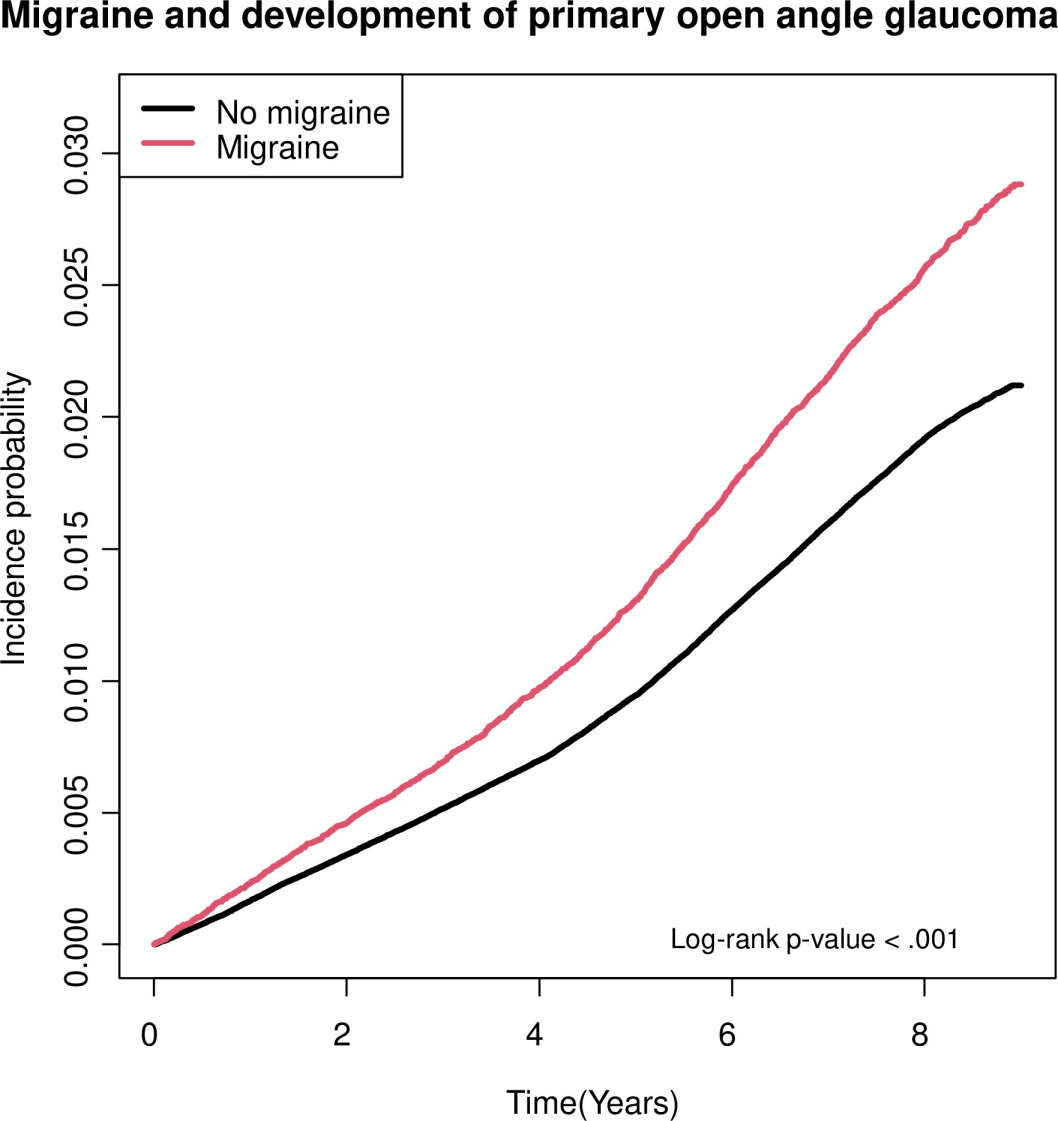
Cumulative incidence of POAG according to migraine diagnosis.

Table 2 shows the incidence and hazard ratio (HR) of POAG according to migraine status. The incidence rate per 1000 person-years was 2.408 and 3.249 in subjects without and with migraine, respectively. Among the migraine group, the incidence rate was 3.137 and 3.893 in mild and severe groups, respectively. The HRs calculated using model-1, model-2, and model-3 were 1.355 (95% confidence interval [CI]: 1.300–1.412), 1.202 (95% CI: 1.153–1.253), and 1.188 (95% CI: 1.140–1.239), respectively. Cumulative incidence of POAG according to history of migraine is illustrated in Fig 2. The log-rank test showed that subjects with migraine had significantly higher POAG development rates compared with those without (*P*< 0.001). In addition, a subgroup analysis was performed to further elucidate the impact of migraine severity on development of POAG. HR was greater when migraine was chronic and severe. HRs for subjects with mild migraine compared to those without migraine were 1.306 (95% CI: 1.248–1.367), 1.181 (95% CI: 1.128–1.236), and 1.169 (95% CI: 1.117–1.224) and those with severe migraine were 1.628 (95% CI: 1.478–1.792), 1.312 (95% CI: 1.191–1.445), and 1.285 (95% CI: 1.166–1.415) when using model-1, model-2, and model-3, respectively.

**Table 2:** The risk of POAG according to the presence and severity of migraine.

		N	Glaucoma	Duration	Incidence rate*	HR(95% C.I)		
						Model 1	Model 2	Model 3
Control		2,628,753	51,354	21,327,443.84	2.408	1 (Ref.)	1 (Ref.)	1 (Ref.)
Migraine		87,809	2,344	721,372.13	3.249	1.355 (1.300,1.412)	1.202 (1.153,1.253)	1.188 (1.140,1.239)
	Non	2,628,753	51,354	21,327,443.84	2.408	1 (Ref.)	1 (Ref.)	1 (Ref.)
	Mild	74,795	1,927	614,250.6	3.137	1.306 (1.248,1.367)	1.181 (1.128,1.236)	1.169 (1.117,1.224)
	Severe	13,014	417	107,121.53	3.893	1.628 (1.478,1.792)	1.312 (1.191,1.445)	1.285 (1.166,1.415)

*per 1000.

Model 1: Non-Adjusted.

Model 2: Adjusted for age, sex, smoking, drinking, exercise, income.

Model 3: Adjusted for age, sex, smoking, drinking, exercise, income, diabetes, hypertension, dyslipidemia, body mass index, and glomerular filtration rate.

We further conducted a risk-stratified analysis to further quantify the impact of migraine in specific patient subgroups. Tables 3 and 4 presents HRs of POAG in subgroups stratified according to subjects’ age, sex, BMI, smoking/drinking habits or presence of diabetes/hypertension/dyslipidemia. The association between migraine and subsequent POAG was consistent in all subgroup analyses. The association of migraine with development of POAG differed according to age (*P* for interaction = 0.004) and HTN (*P* for interaction = 0.001) (Table 3). The impact of migraine on POAG development was greater in the younger group (aHR: 1.235, 95% CI: 1.168–1.305) compared to those aged 65 or older (aHR: 1.139, 95% CI: 1.069–1.214). In addition, the aHR of POAG diagnosis during the 9-year follow-up period was greater in those without hypertension (aHR: 1.261, 95% CI:1.186–1.341) compared to those with hypertension (aHR: 1.128, 95% CI:1.066–1.194) Similar pattern was noted when subjects with chronic and severe migraine were classified into a subgroup (Table 4).

**Table 3:** Risk of POAG according to presence of migraine stratified by age, sex, body mass index, diabetes, hypertension, dyslipidemia, smoking, and drinking.

Subgroup		Migraine	N	POAG	Duration	Incidence Rate*	Model	p for interaction
Age								
	Age < 65	No	2,149,553	32,198	17,683,623.25	1.821	1 (Ref.)	0.004
		Yes	65,394	1,323	549,220.28	2.409	1.235 (1.168,1.305)	
	Age ≥ 65	No	479,200	19,156	3,643,820.59	5.257	1 (Ref.)	
		Yes	22,415	1,021	172,151.85	5.931	1.139 (1.069,1.214)	
Sex								
	Male	No	1,343,046	25,792	10,797,589.06	2.389	1 (Ref.)	0.676
		Yes	24,289	683	194,793.27	3.506	1.203 (1.115,1.298)	
	Female	No	1,285,707	25,562	10,529,854.79	2.428	1 (Ref.)	
		Yes	63,520	1,661	526,578.86	3.154	1.181 (1.123,1.241)	
BMI								
	BMI <25	No	1,709,956	32,465	13,841,661.84	2.345	1 (Ref.)	0.851
		Yes	56,828	1,473	465,593.08	3.164	1.192 (1.131,1.256)	
	BMI ≥25	No	918,797	18,889	7,485,782.01	2.523	1 (Ref.)	
		Yes	30,981	871	255,779.05	3.405	1.182 (1.104,1.266)	
Diabetes								
	No	No	2,325,267	41,500	18,949,713.72	2.190	1 (Ref.)	0.134
		Yes	77,973	1,933	644,174.72	3.001	1.197 (1.143,1.253)	
	Yes	No	303,486	9,854	2,377,730.13	4.144	1 (Ref.)	
		Yes	9,836	411	77,197.41	5.324	1.152 (1.043,1.272)	
Hypertension								
	No	No	1,709,603	25,915	14,007,504.29	1.850	1 (Ref.)	0.001
		Yes	50,294	1,080	418,650.89	2.580	1.261 (1.186,1.341)	
	Yes	No	919,150	25,439	7,319,939.55	3.475	1 (Ref.)	
		Yes	37,515	1,264	302,721.24	4.175	1.128 (1.066,1.194)	
Dyslipidemia								
	No	No	2,033,337	35,914	16,515,739.76	2.175	1 (Ref.)	0.387
		Yes	62,309	1,494	512,885.85	2.913	1.204 (1.143,1.268)	
	Yes	No	595,416	15,440	4,811,704.08	3.209	1 (Ref.)	
		Yes	25,500	850	208,486.28	4.077	1.161 (1.083,1.245)	
Current Smoking								
	No	No	2,066,344	43,046	16,804,046.02	2.562	1 (Ref.)	0.085
		Yes	78,348	2,125	645,083.42	3.294	1.178 (1.127,1.23)	
	Yes	No	562,409	8,308	4,523,397.82	1.837	1 (Ref.)	
		Yes	9,461	219	76,288.71	2.871	1.296 (1.132,1.483)	
Drinking								
	No	No	1,521,777	3,3091	12,319,670.94	2.686	1 (Ref.)	0.857
		Yes	64,916	1,842	532,478.19	3.459	1.187 (1.133,1.244)	
	Yes	No	1,106,976	18,263	9,007,772.91	2.027	1 (Ref.)	
		Yes	22,893	502	188,893.94	2.658	1.191 (1.09,1.302)	

*per 1000.

Model: Adjusted for age, sex, smoking, drinking, exercise, income, diabetes, hypertension, dyslipidemia, body mass index, and glomerular filtration rate.

**Table 4:** Risk of POAG according to migraine severity stratified by age, sex, BMI, diabetes, hypertension, dyslipidemia, smoking, and drinking.

Subgroup	Migraine	N	POAG	Duration	Incidence Rate*	Model	p for interaction
**Age**							
Age < 65	Control	2,149,553	32,198	17,683,623.25	1.821	1(Ref.)	0.001
	Mild	56,596	1,096	474,541.2	2.310	1.193(1.123,1.268)	
	Severe	8,798	227	74,679.07	3.040	1.486(1.304,1.693)	
Age ≥ 65	Control	479,200	19,156	3,643,820.59	5.257	1(Ref.)	
	Mild	18,199	831	139,709.39	5.948	1.141(1.064,1.223)	
	Severe	4,216	190	32,442.46	5.857	1.132(0.981,1.306)	
**Sex**							
Male	Control	1,343,046	25,792	10,797,589.06	2.389	1(Ref.)	0.689
	Mild	21,759	598	174,939.09	3.418	1.199(1.105,1.3)	
	Severe	2,530	85	19,854.18	4.281	1.232(0.996,1.525)	
Female	Control	1,285,707	25,562	10,529,854.79	2.428	1(Ref.)	
	Mild	53,036	1,329	439,311.51	3.025	1.155(1.093,1.22)	
	Severe	10,484	332	87,267.35	3.804	1.297(1.164,1.446)	
**BMI**							
BMI < 25	Control	1,709,956	32,465	13,841,661.84	2.345	1(Ref.)	0.625
	Mild	48,387	1,203	396,339.51	3.035	1.165(1.099,1.234)	
	Severe	8,441	270	69,253.57	3.899	1.331(1.18,1.5)	
BMI ≥ 25	Control	918,797	18,889	7,485,782.01	2.523	1(Ref.)	
	Mild	26,408	724	217,911.08	3.322	1.177(1.093,1.268)	
	Severe	4,573	147	37,867.96	3.882	1.208(1.027,1.422)	
**Diabetes**							
No	Control	2,325,267	41,500	18,949,713.72	2.190	1(Ref.)	0.171
	Mild	66,563	1,587	549,580.18	2.888	1.173(1.116,1.234)	
	Severe	11,410	346	94,594.54	3.658	1.32(1.187,1.468)	
Yes	Control	303,486	9,854	2,377,730.13	4.144	1(Ref.)	
	Mild	8,232	340	64,670.42	5.257	1.152(1.034,1.284)	
	Severe	1,604	71	12,526.99	5.668	1.15(0.91,1.453)	
**Hypertension**							
No	Control	1,709,603	25,915	14,007,504.29	1.850	1(Ref.)	0.005
	Mild	43,456	903	361,271.5	2.500	1.243(1.163,1.328)	
	Severe	6,838	177	57,379.38	3.085	1.364(1.176,1.582)	
Yes	Control	919,150	25,439	7,319,939.55	3.475	1(Ref.)	
	Mild	31,339	1,024	252,979.09	4.048	1.107(1.039,1.178)	
	Severe	6,176	240	49,742.15	4.825	1.232(1.085,1.399)	
**Dyslipidemia**							
No	Control	2,033,337	35,914	16,515,739.76	2.175	1(Ref.)	0.576
	Mild	53,693	1,241	441,698.96	2.810	1.182(1.116,1.251)	
	Severe	8,616	253	71,186.88	3.554	1.325(1.171,1.5)	
Yes	Control	595,416	15,440	4,811,704.08	3.209	1(Ref.)	
	Mild	21,102	686	172,551.63	3.976	1.147(1.062,1.238)	
	Severe	4,398	164	35,934.65	4.564	1.227(1.052,1.432)	
**Current Smoking**							
No	Control	2,066,344	43,046	16,804,046.02	2.562	1(Ref.)	0.203
	Mild	66,314	1,737	545,801.99	3.182	1.157(1.103,1.214)	
	Severe	12,034	388	99,281.43	3.908	1.279(1.157,1.414)	
Yes	Control	562,409	8,308	4,523,397.82	1.837	1(Ref.)	
	Mild	8,481	190	68,448.61	2.776	1.284(1.112,1.484)	
	Severe	980	29	7,840.1	3.699	1.378(0.957,1.985)	
**Drinking**							
No	Control	1,521,777	33,091	12,319,670.94	2.686	1(Ref.)	0.461
	Mild	54,216	1,495	444,426.48	3.364	1.172(1.113,1.235)	
	Severe	10,700	347	88,051.7	3.941	1.257(1.131,1.398)	
Yes	Control	1,106,976	18,263	9,007,772.91	2.027	1(Ref.)	
	Mild	20,579	432	169,824.11	2.544	1.158(1.052,1.274)	
	Severe	2,314	70	19,069.83	3.671	1.452(1.148,1.836)	

*per 1000.

Model: Adjusted for age, sex, smoking, drinking, exercise, income, diabetes, hypertension, dyslipidemia, body mass index, and glomerular filtration rate.

## Discussion

We found that subjects with migraine were independently associated with a 1.19 times increased risk of POAG diagnosis after adjusting for age, sex, smoking, drinking, exercise, income, diabetes, hypertension, dyslipidemia, body mass index, and glomerular filtration rate within 9 years after their diagnosis. While those with mild migraine showed 1.17 times greater risk, those with severe and chronic migraine showed 1.29 times greater risk of POAG after adjusting for confounding factors.

Migraine is an episodic neurologic disorder characterized by recurrent attacks of throbbing headache and typical premonitory features such as nausea or sensory hypersensitivity. The development of symptoms are related with the activation of trigeminovascular system and cortical spreading depression, although underlying mechanisms are still up for discussion [5]. It is noteworthy that glaucoma shares certain characteristics with migraine in that both are possibly associated with vascular dysregulation in terms of pathophysiology of the diseases [29, 30]. Moreover, vasospasm intensity, calculated based on the presence or absence of migraine, cold extremities and vasospastic response to temperature change, is strongly associated with the development of the disease in particular subgroups of glaucoma [31]. Broadway and Drance [31] discovered that glaucoma patients with focal ischemia of the optic nerve head and subsequent focal loss of the neuroretinal rim had a higher prevalence of vasospasm and migraine than glaucoma patients without these forms of optic tissue destruction. Whether migraines can significantly increase the risk of developing POAG is still controversial. For example, Lin et al. [32]. reported that subjects with migraine were more likely to have POAG compared with those without migraine, even after adjusting for gender, age, monthly income, and level of urbanization of the community. The Collaborative Normal-Tension Glaucoma Study Group analyzed the risk factors for deterioration of visual field defects in NTG and found migraine to be an independent risk factor for more rapid progression [11]. Huang et al. [33] found that migraine is associated with a higher risk of open angle glaucoma in patients with no comorbidity who are aged under 50 years. Motsko et al. reported migraines were found to be independent risk factors for the development of OAG [34]. Also, other previous studies found significant association between migraine and POAG [35, 36]. On the other hand, some studies reported that migraines did not increase the risk of POAG [37–39]. Landers et al. found there was no significant association between migraine and POAG after adjusting for confounders [39]. We believe that diverse study designs and ethnic populations may be contributing to the disparities among studies.

Prevalence of migraine and POAG both increase along with the subjects’ age. It was consistently reported that migraine remained to be a significant risk factor for the development of POAG when the relative risk was adjusted for the subjects’ age [27, 33]. To corroborate this observation, we performed a risk-stratified analysis in our study (Tables 3 and 4). Notably, when the subjects were divided into two age groups, the impact of migraine on the development of POAG was greater in those who were younger than 65 compared to those aged 65 or older. In addition, we also found that migraineurs without comorbid hypertension showed higher risk of POAG than those with hypertension. Further research is warranted to clarify our findings.

Previously, Huang et al. [33] also reported that the cumulative incidence of POAG was significantly higher among the migraineurs compared to normal control subjects. However, because it was not possible to perform a subclassification of migraine group in their study, the relationship between glaucoma progression and migraine severity was not addressed. To shed light on this, we subclassified subjects with migraine based on chronicity and severity of migraine based on the frequency and type of insurance claims. It is worth to note that we saw stronger evidence of association between migraine and POAG, when the course of migraine was worse (Table 2).

Our study has the following strengths. First, the sample size is large enough to jointly evaluate the impact of migraine on the development of POAG, with the effect of other variables including age, sex, smoking or drinking habits, socioeconomic status, and comorbid diseases. In addition, we aimed to establish a temporal and causal relationship between migraine and POAG onset using this longitudinal database by excluding those previously diagnosed with POAG before or within one year after the index year. Secondly, we only included those with medically diagnosed migraine and POAG rather than self-questionnaires, thereby increasing the validity of the study subjects. Thirdly, although confined to Koreans, the study population of the present study well represents the actual population composition of the entire country, given that it is an obligation to join the KNHIS for all individuals in South Korea.

Our study also has some limitations. All data were collected from KHED and NHID, which originally were not established with the sole intention of being used for research purpose which might have led to some limitations. First, people with undiagnosed glaucoma were not included in our estimates of glaucoma prevalence, which may have resulted in an underestimated prevalence. Second, national claims data do not always match hospital chart records. It has been shown that national claims data and hospital records only have a 70–80% concordance rate [40]. We attempted to mitigate this by including only those with at least three visits under the diagnosis of glaucoma, but this may have affected our results. Third, we haven’t excluded or controlled for different classes of antihypertensive medication which can be associated with glaucoma development or progression. Some studies found that antihypertensive drugs seem to reduce the risk of developing glaucoma [41]. On the other hand, others found that antihypertensive medication compromises optic nerve head blood flow which may lead to a higher risk of glaucomatous progression [42, 43]. Finally, the degree of migraine can vary depending on the medication, however, we have not included the effect of migraine medication on chronicity and severity of migraine in the subanalysis. Also, the clinical information about glaucoma medication, glaucomatous optic neuropathy and visual field defects were unavailable and measurement and adjustment for factors a priori known to be associated with the POAG, such as intraocular pressure, might strengthen the importance of our findings.

## Conclusion

In conclusion, our study suggests that presence and severity of migraine are both associated with increased risk of subsequent development of POAG. We found that the hazard of POAG development was 1.19 times greater in subjects with migraine compared to those without after adjusting for age, sex, lifestyle factors, and comorbidities. Furthermore, we also found that subjects with chronic and severe migraine showed 1.29 times greater hazard of POAG development. It is recommended that neurologists refer migraineurs for glaucoma assessment, especially when migraine is chronic and severe.
